# Influence of Different Pretreatments on the Antibacterial Properties of Chitosan Functionalized Viscose Fabric: TEMPO Oxidation and Coating with TEMPO Oxidized Cellulose Nanofibrils

**DOI:** 10.3390/ma12193144

**Published:** 2019-09-26

**Authors:** Matea Korica, Zdenka Peršin, Snežana Trifunović, Katarina Mihajlovski, Tanja Nikolić, Slavica Maletić, Lidija Fras Zemljič, Mirjana M. Kostić

**Affiliations:** 1 Innovation Center of Faculty of Technology and Metallurgy, University of Belgrade, Karnegijeva 4, 11000 Belgrade, Serbia; mkorica@tmf.bg.ac.rs; 2 Institute of Engineering Materials and Design, Faculty of Mechanical Engineering, University of Maribor, Smetanova ulica 17, 2000 Maribor, Slovenia; zdenka.persin@um.si (Z.P.); lidija.fras@um.si (L.F.Z.); 3 Faculty of Chemistry, University of Belgrade, Studentski trg 12-16, 11000 Belgrade, Serbia; snezanat@chem.bg.ac.rs; 4 Faculty of Technology and Metallurgy, University of Belgrade, Karnegijeva 4, 11000 Belgrade, Serbia; kmihajlovski@tmf.bg.ac.rs (K.M.); tanjanikol@gmail.com (T.N.); 5 Faculty of Physics, University of Belgrade, Studentski trg 12-16, 11000 Belgrade, Serbia; sslavica@ff.bg.ac.rs

**Keywords:** viscose, chitosan, TEMPO oxidation, TEMPO oxidized cellulose nanofibrils, antibacterial properties

## Abstract

The main objective of this study was to obtain chitosan functionalized viscose fabric with improved antibacterial properties and washing durability. In this regard carboxyl and aldehyde groups, as binding points for irreversible chitosan attachment into/onto viscose fabric, were introduced by two different pretreatments: 2,2,6,6-tetramethylpiperidine-1-oxy radical (TEMPO) oxidation and coating with TEMPO oxidized cellulose nanofibrils (TOCN). The Fourier transform infrared spectroscopy, elemental analysis, zeta potential measurements, scanning electron microscopy, breaking strength and antibacterial testing were used to evaluate the influence of these pretreatments on chitosan binding, but also on chemical, electrokinetic, morphological, mechanical and antibacterial properties of pretreated and chitosan functionalized viscose fabrics. Washing durability of chitosan functionalized viscose was monitored through changes in the chitosan content, electrokinetic and antibacterial properties after multiple washing. TOCN coating improves mechanical properties of fabric, while TEMPO oxidation deteriorates them. The results show that both pretreatments improve chitosan adsorption and thus antibacterial properties, which are highly durable to washing. After five washings, the chitosan functionalized pretreated viscose fabrics preserve their antibacterial activity against *Staphylococcus aureus*, while antibacterial activity against *Escherichia coli* was lost. TOCN coated and chitosan functionalized viscose fabric is a high value-added product with simultaneously improved antibacterial and mechanical properties, which may find application as medical textiles.

## 1. Introduction

The great potential of viscose as a material for medical textiles lies in its molecular structure, which offers excellent possibilities as a matrix for the design of biocompatible, bioactive and high performance materials [1]. With increasing awareness of infectious diseases and antibiotic resistance, many studies were dedicated to the functionalization of textiles, which would assure antibacterial activity together with complete safety for the user [2]. All of this paves the way for the development of healthcare textiles. Textile materials for healthcare and medical products range from simple bandage materials or gauze, to scaffolds for tissue repair and a large variety of prostheses for body implants. The use of medical textiles depends on their properties, such as flexibility, absorption, softness and filtering, among others [3]. Medical textiles are now being used both in the medical and biological sector. They are used for first aid, rehabilitation, hygienic or clinical purposes. In most of these cases, natural fibers with stable bioactive efficiency and good mechanical properties are required. Traditionally, the antimicrobial protection of textiles included many chemical substances, such as inorganic salts, phenols, antibiotics, iodine derivatives, nitro compounds, formaldehyde derivatives, amines, etc. [4]. Unfortunately, the drawback of these compounds is their toxicity, along with poor biodegradability, which is unacceptable from the point of view of current environmental and health protection legislation. Beside these, the fact that consumers are becoming increasingly aware of the need to embrace healthier life styles is another reason why textile functionalization has been forced to involve natural, non-toxic active substances in the technological process, thus causing minimal or no side-effects to people as well as to the environment [5].

In this regard, functionalization is performed by the use of non-toxic, biodegradable and environmentally friendly reagents, which include alternative and less used polysaccharides and their derivatives such as chitosan (CS) [6]. Various procedures for viscose functionalization with CS have been presented until now [1,7,8,9,10,11,12,13]; however, attention has not been paid to washing durability of viscose functionalized with CS, to the best of our knowledge. For obtaining textiles with washing durable antibacterial activity, efficient and permanent fixation of CS into/onto viscose is of major importance.

Similarities between the chemical and molecular structures of cellulose and CS enable high affinity between the two polymers. The most likely cellulose–chitosan intermolecular interactions are based on H-bonds and Van der Waals forces. However, for more intense and irreversible binding between cellulose and CS, it is necessary to introduce carboxyl and/or aldehyde groups into/onto the cellulose, which are then potential anchoring sites for CS molecules [14]. Carboxylic groups of cellulose as host material can provide electrostatic attraction between them and CS as adsorbate, while aldehyde groups are an excellent choice because they can allow covalent binding of CS by forming a Schiff base [1]. These bounds, when present at the same time, may serve as a starting point for irreversible binding of CS onto cellulose [14].

Carboxylic and aldehyde groups can be introduced into cellulose by numerous chemical modifications [15]. Oxidation, the only process that renders cellulose bioabsorbable in humans, is the most widely used, but usually leads to deterioration of the mechanical properties of the cellulose substrate [16,17,18,19]. In recent years, the N-oxyl radical-mediated oxidation using, for example, 2,2,6,6-tetramethylpiperidine-1-oxy radical (TEMPO) was found to be the most promising reaction for efficient conversion of primary hydroxyl groups to carboxylates via aldehydes or as a pretreatment step in nanocelulose production [18]. In design and production of novel biomedical materials, the nanocellulose has gained much attention because of its remarkable physical properties, special surface chemistry and excellent biological properties (biocompatibility, biodegradability and low toxicity). Nanocellulose is a unique and promising natural material extracted from native cellulose, suitable for modification of various materials in order to improve existing or give completely new properties [18,19,20,21,22]. By TEMPO oxidation of natural cellulose fibers and successive mechanical and/or ultrasound treatments, nanofibrillated cellulose with an increased content of carboxyl and aldehyde groups, the so-called TEMPO oxidized cellulose nanofibrils (TOCN), are obtained. Furthermore, biodegradability/biological stability and hydrophilic/hydrophobic properties of fibrous TEMPO oxidized cellulose and TOCN can be tailored by controlling carboxyl group content and their counterions [18,21]. In view of these facts, TOCN makes a very favorable substrate for coating textile materials based on cellulose, allowing the introduction of a high amount of functional groups (carboxyl and aldehyde) on their surface without deterioration of mechanical properties, and thereafter providing a stable CS adsorption [14,21,22,23]. According to a detailed literature review and the best of our knowledge, the pretreatments such as TEMPO oxidation as well as coating of viscose materials with TOCN followed by functionalization with CS and characterization of the product were not reported until now.

In the present work, in order to improve antibacterial properties and washing durability of chitosan functionalized viscose fabric, these two pretreatments, i.e., TEMPO oxidation under neutral conditions and coating with TOCN obtained by defibrillation of TEMPO oxidized cotton fibers, were used to introduce carboxyl and aldehyde groups, which are necessary for irreversible binding of CS. The influences of these pretreatments on the chemical, electrokinetic, morphological, mechanical and antibacterial properties of pretreated and CS functionalized fabrics were investigated and compared. The fabrics were characterized using the Fourier transform infrared spectroscopy (FTIR), elemental analysis of nitrogen, carboxyl and aldexyde groups content, zeta potential measurements, scanning electron microscopy (SEM), breaking strength testing and antibacterial testing under dynamic contact conditions. Washing durability of chitosan functionalized viscose fabrics was monitored through changes in the nitrogen content, electrokinetic surface properties and antibacterial activity after one, three and five washing cycles.

## 2. Materials and Methods 

### 2.1. Materials

Regenerated cellulose fabric, (15A23 viscose uni Sandy–white), as provided by IGR Agence was used as a base material. The surface mass of the fabrics was 82 g/m^2^, yarn count of 9.6 tex × 9.9 tex, fabric count of 400 warp threads/10 cm and 350 weft threads/10 cm. Chitosan (CS) from crab shells with low molecular weight (Aldrich, 448869), 75%–85% deacetylated, purchased from Sigma-Aldrich (Vienna, Austria). TEMPO, sodium bromide (NaBr), sodium hypochlorite (NaClO), sodium chlorite (NaClO_2_), sodium hydroxide (NaOH) and 13% sodium hypochlorite (NaClO) solution were from Sigma-Aldrich (Vienna, Austria), and applied without further purification.

### 2.2. Preparation of the Chitosan Solution 

A 0.5% solution of CS was prepared by suspending CS in distilled water, afterwards setting the pH to 2.5 with the use of hydrochloric acid (1 M HCl). The resulting suspension was then stirred for 24 h using a laboratory magnetic stirrer under room temperature until achieving a complete dissolution of the CS. The final pH of the CS solution was adjusted to pH 5.5 with 0.5 M NaOH.

### 2.3. Preparation of TEMPO Oxidized Cellulose Nanofibrils 

Cotton fibers (Russian, I class, 32/33 mm) were pretreated with NaClO, catalytic amounts of TEMPO and sodium bromide, according to a method described elsewhere [24]. In a typical experiment, 10 g of cotton fibers were suspended in water (750 mL) containing TEMPO (0.0250 g) and sodium bromide (0.250 g). A designated amount of the 13 wt% NaClO solution, corresponding to 15 mmol/g cellulose, was added slowly to the cellulose slurry under continuous stirring. The pH of the slurry at room temperature was maintained at 10.5 by addition of 0.5 M NaOH during 3 h. The oxidation was quenched by adding ethanol (ca. 5 mL). The oxidized cellulose was washed thoroughly with distilled water, subsequently with ethanol and lastly with distilled water on a filter paper set in a Büchner funnel. The TEMPO oxidized cellulose nanofibrils with sodium carboxylate groups (TOCN-COONa) dispersed in water were obtained by passing a slurry of TEMPO-oxidized cotton fibers in water (100 mL, 0.5% solid consistency) through a double cylinder-type homogenizer (T 25 digital ULTRA-TURRAX, IKA, Staufen, Germany) for 5 min at 1000 rpm, and its subsequent sonication for 15 min using an ultrasonic homogenizer (WCX 750, SONICS, Newtown, Australia) with a 19 mm diameter probe tip at 20 kHz and 750 W output power.

### 2.4. Pretreatment of Viscose Fabric 

In order to promote better adsorption of CS onto viscose, viscose fabric was modified to introduce carboxyl and aldehyde groups as binding sites for amino groups of CS, by two different pretreatments:

(a) TEMPO oxidation carried out under neutral conditions (pH 7) according to a method described previously [25]. Viscose fabric (10 g) was soaked in a 0.05 M sodium phosphate buffer solution at pH 7 (50 mL phosphate buffer solution/g viscose fabric), containing TEMPO (20 mg TEMPO/g viscose fabric). Sodium chlorite (50 mg NaClO_2_/g viscose fabric) and sodium hypochlorite solution (2.5 mmol NaClO/g viscose fabric) were added to the flask and stirred at 200 rpm and 60 °C for 5 min. After stirring, the oxidation was quenched by adding ethanol (ca 5 mL). The oxidized viscose fabric was thoroughly washed with distilled water by filtration, subsequently with ethanol, and dried at room temperature.
(b) Coating of viscose fabrics with 0.5% (w/v) dispersion of TOCN: The treatment lasted for 30 min at room temperature using a material-liquid bath ratio of 1:50, with a wet pick-up of 100%. Samples were then squeezed onto a laboratory padder (Rapid, Istanbul, Turkey) at a pressure of 2 bars. After the excess liquid was removed, fabric was dried at 40 °C for 30 min in a laboratory oven (Instrumentaria, Zagreb, Croatia).

Both pretreated samples were then conditioned (T = 20 ± 2 °C; RH = 65% ± 4%) before being functionalized with CS.

### 2.5. Functionalization with Chitosan

The pretreated and pristine viscose fabrics were immersed into the aqueous 0.5% CS solution. The treatment lasted for 30 min at room temperature using a material–liquid bath ratio of 1:50, with a wet pick-up of 100%. Samples were then squeezed onto a laboratory padder (Rapid, Istanbul, Turkey) at a pressure of 2 bars. After the excess liquid was removed, fabrics were dried at 40 °C for 30 min in a laboratory oven (Instrumentaria, Zagreb, Croatia). Fabrics were then conditioned (T = 20 ± 2 °C; RH = 65% ± 4%) before being analyzed further.

### 2.6. Washing of Functionalized Viscose Fabrics

Washing of functionalized viscose fabrics was performed according to standard ISO 105-C10. The samples were washed in a bath containing 0.5% standard soap at 40 °C for 30 min. After washing, samples were rinsed with distilled water for 1 min and then thoroughly rinsed with tap water for 1 min, and dried at 40 °C in a laboratory oven (Instrumentaria, Zagreb, Croatia).

The sample notations of viscose fabrics before and after washing are presented in Table 1.

### 2.7. The Carboxyl Group Content

The carboxyl groups of cellulose react with the salts of weaker acids, such as calcium acetate, forming a salt of the cellulose and releasing an equivalent amount of the weaker acid. Based on that and a modification of the calcium-acetate method [26] for determining the carboxyl content in viscose fabrics and TEMPO oxidized cotton fibers, the following method was applied. Fabrics/fibers (0.5 g) were treated with 0.01 M HCl for 1 h and then washed thoroughly with distilled water. In the next step, 50 mL of distilled water and 30 mL 0.25 M of calcium acetate solution were added to the fabrics. After standing for 2 h with frequent shaking to facilitate completion of the interchange, 30 mL portions of the liquid were titrated with 0.01 M sodium hydroxide, using phenolphthalein as an indicator. The carboxyl content is calculated as follows:

(1) $$COOH=\frac{\frac{80}{30}\cdot 0.01M\cdot V\left(NaOH\right)}{m\cdot \left(1-\frac{w}{100}\right)},\mathrm{mmol}/g,$$

where 0.01 M is the concentration of NaOH, *V*(NaOH) is the volume (mL) of the NaOH solution used for titration, *m* is the weight of the fabrics/TEMPO oxidized cotton fibers (g) and *w* is the moisture content (%).

### 2.8. The Aldehyde Group Content

The aldehyde content in fabrics/fibers was determined according to the modified method described by Parks and Hebert [26,27]. The aldehyde groups of the samples were oxidized selectively to carboxyl groups with sodium chlorite at pH 4–5, and the carboxyl content was determined by the above mentioned calcium-acetate method. The fabrics/TEMPO oxidized cotton fibers (1 g) were added to a mixture containing NaClO_2_ (0.905 g), 5 M CH_3_COOH (10 mL) and distilled water (50 mL). Oxidation was carried out by stirring the mixture at room temperature for 48 h, followed by washing with water and filtration. The carboxyl groups formed by the NaClO_2_ oxidation were regarded as aldehyde groups present in fabrics/TEMPO oxidized cotton fibers. Those groups were determined as pointed out above.

### 2.9. FTIR Analysis

Attenuated total reflectance accessory (ATR)-FTIR spectra of the samples were recorded using Shimadzu IRA Infinity-1 (FT-IR) spectrophotometer (Shimadzu Corporation, Kyoto, Japan) equipped with attenuated total reflectance accessory (ATR) using a diamond/ZnSe crystal in the wavenumber range of 600–4000 cm^−1^, at a resolution of 2 cm^−1^ and in 20 scan mode. Before ATR-FTIR measurements, samples were dried overnight at 40 °C and stored in the desiccator until the measurements.

### 2.10. Elemental Analyses

The chitosan content in the functionalized viscose fabrics was calculated from the nitrogen percentage, determined by the elemental analysis performed on a Vario EL III C,H,N,S/O Elemental Analyzer (Elementar Analysensysteme GmbH, Langenselbold, Germany).

### 2.11. The Streaming Potential Measurement

The zeta potential of viscose fabric was determined by the streaming potential method using a SurPASS Electrokinetic Analyzer (Anton Paar GmbH, Graz, Austria). A rectangular fabric sample (8 cm × 2 cm), was mounted in the cylindrical cell, thereby creating a permeable plug. The reproducible packing density of the fabric plug was maintained by monitoring the sample size and weight and controlling the sample compression in the measuring cell. To avoid the influence of the substrate swelling on the zeta potential, samples were pre-swelled in distilled water for 30 min before measurement. A 0.001 M KCl solution was used as the electrolyte and the initial pH was adjusted to pH 10 with NaOH, while, during automatic titrations, changes in pH (from about pH 10 to pH 3) were achieved by the addition of 0.05 M HCl. Since the ionic strength of an electrolyte solution during measurement in a low pH region can interfere with the zeta potential [28], measurements were carried down to pH 3, and isoelectric points (IEP) lower than 3 were determined by extrapolation of experimental data. Four measurements were performed for each sample and standard deviation was up to 5%.

### 2.12. SEM Analysis

The morphological properties of viscose fibers were investigated using a JEOL JSM-5300 scanning electron microscope (JEOL, Tokyo, Japan). Prior to analysis the samples were sputtered with gold ION SPUTTER, JEOL, model JFC-1100E (JEOL, Tokyo, Japan).

### 2.13. Breaking Strength Testing

Breaking strength of viscose fabrics was determined according to standard SRPS EN ISO 13934-1:2008, using a standard fabric constant-rate-of-loading machine (Textest, Schwerzenbach, Switzerland), with clamps spaced at 200 mm, and with strain rate (bottom clamp rate) of 150 mm/min. The width of test strips was 50 mm ± 0.5 mm (excluding any fringe). According to standard SRPS EN ISO 139:2007, prior to measurements, the samples were conditioned in the relaxed state, and tested under the standard atmosphere (T = 20 ± 2 °C; RH = 65% ± 4%).

### 2.14. Antibacterial Testing

The antimicrobial activity of fabrics was tested against Gram-negative bacteria *Escherichia coli* ATCC 25922 (*E. coli*) and Gram-positive bacteria *Staphylococcus aureus* ATCC 25923 (*S. aureus*), using a standard test method for determining the antimicrobial activity of immobilized antimicrobial agents under dynamic contact conditions ASTM E 2149-01 (2001). Prior to investigation, the samples were UV sterilized. The microorganisms were grown overnight in tryptone soy broth (TSB, Torlak, Serbia) at 37 °C, supplemented with 0.6% Yeast Extract (Torlak, Serbia). The sterile physiological saline solutions (9 mL) were inoculated with 1 mL of inoculum-test strains, providing the initial concentration of ~10^5^ colony forming units (CFU) per mL. The pristine viscose fabric (control) and chitosan functionalized viscose fabrics (0.2 g) cut into small pieces were added in the flask and they were incubated at 37 °C for 24 h. After a series of dilutions, 1 mL aliquot of the solution was transferred in the sterile Petri dish covered with tryptone soy agar. The inoculated plates were incubated at 37 °C for 24 h and surviving cells were counted. The percentage of bacterial reduction (*R*, %) was calculated by the following equation:

(2) $$R=\frac{C_{0}-C}{C_{0}}\times 100\%,$$

where *C*_0_ (CFU) is the number of bacteria colonies on the control sample and *C* (CFU) is the number of bacteria colonies on the investigated fabric.

## 3. Results and Discussion

### 3.1. Characterization of Differently Pretreated and Functionalized Viscose Fabrics

#### 3.1.1. Carboxyl and Aldehyde Group Content

Two different pretreatments were used in order to introduce carboxylic and/or aldehyde functional groups into/onto viscose fabrics (Table 2), since the presence of these functional groups should contribute to irreversible binding of chitosan. Conversion of the primary hydroxyl groups of glucopyranose units into carboxyl groups through aldehyde intermediates during TEMPO oxidation leads to an increase in the functional groups content of TEMPO oxidized viscose, as well as TOCN. Coating pristine viscose fabric with TOCN, whose surface is decorated with a high content of functional groups, enables surface material decoration with carboxyl and aldehyde groups and contributes to a slight increase in the content of both functional groups in the viscose fabric. Compared to pristine, an increase in functional group content (carboxyl + aldehyde) of 10.7 times was achieved for TEMPO oxidized fabrics, and of 1.4 times for TOCN coated viscose fabrics.

#### 3.1.2. FTIR and Elemental Analyses

**Figure 1:**
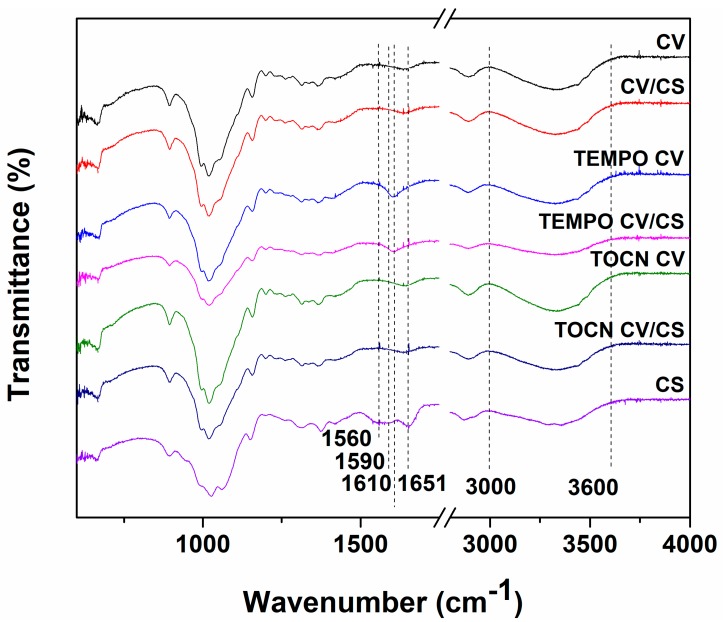
FTIR spectra of viscose fabrics before and after chitosan functionalization.

The FTIR-ATR technique was used as a straightforward method to characterize the surface of the viscose fabrics before and after different pretreatments and chitosan functionalization (Figure 1). In the FTIR spectra of the pristine and differently pretreated viscose fabrics, the characteristic bands of cellulose can be observed: The bands at 892–896 cm^−1^ (C–O–C valence vibration of the β-glycosidic linkage or deformation at C(1) in cellulose II), the band at 996 cm^−1^ (C–O valence vibration at C(6)), the bands at 1024 and 1067 cm^−1^ (skeletal vibrations involving C–O stretching), the band at 1158 cm^−1^ (C–O–C asymmetric from β-glycosidic link in cellulose II), the bands at 1200 and 1336 cm^−1^ (OH in-plane bending vibrations), the band at 1225–1235 cm^−1^ (O–H in-plain deformation at C(6)), the band at 1316 cm^−1^ (CH_2_ wagging vibration), the band at 1372 cm^−1^ (C–H deformation in cellulose II), the band at 1418 cm^−1^ (CH_2_ scissoring at C(6) in cellulose II), the broad band at 1636 cm^−1^ (adsorbed water), the band at 2893 cm^−1^ (C–H stretch in cellulose II and CH_2_, CH_2_OH in cellulose from C6) and the broad band between 3000–3600 cm^−1^ (hydrogen-bonded OH groups) [29,30]. After TEMPO oxidation, the appearance of a new band in the spectra of TEMPO CV fabric at 1600 cm^−1^, attributed to the C=O stretching vibration, confirms that hydroxyl groups at the C6 position of cellulose molecules were converted to sodium carboxylate [31,32]. The absence of the absorption band at 1600 cm^−1^ in the spectra of TOCN CV fabric can be explained by a low content of the carboxylate groups in this sample (Table 2), but also by the fact that this absorption band is in a region of the spectra where the broad band of adsorbed water could mask its signal. Furthermore, the aldehyde group estimation appears to be difficult since these groups exist partially or even totally hydrated, and the resulting hemiacetal or hemialdol structures do not exhibit the classical adsorption of the carbonyl group in the FTIR spectra [32]. In these conditions, the aldehyde group originating from TEMPO oxidation appears to be hardly detected by FTIR. However, in the case of TEMPO CV and TOCN CV fabrics, a slight decrease in the intensity of the bands at 996, 1225–1235 and 1418 cm^−1^, corresponding to C–O valence vibration at C(6), O–H in-plane deformation at C(6), and CH_2_ scissoring at C(6) in cellulose II, respectively, can be observed.

The FTIR spectra of pure chitosan and differently pretreated viscose fabrics, before and after functionalization with chitosan, are also presented in Figure 1. The FTIR spectrum of CS shows the main absorption bands of pure chitosan: The bands at 894 and 1153 cm^−1^ correspond to a saccharide structure, similar to cellulose; the bands at 1025 and 1062 cm^–1^ related to C–O stretching; the band at 1315 cm^−1^ reported as the amide III peak; the bands at 1373 and 1420 cm^−1^ assigned to the C–Hx deformations; the band at 1560 cm^−1^ assigned to the stretching vibration of the amino group of chitosan; the band at 1590 cm^−1^ related to the N–H bending of the amide II; the band at 1651 cm^−1^ reported as the amide I peak overlaps with the water absorbance peak; the bands at 2870 and 2921 cm^-1^ attributed to C–H asymmetric and symmetric stretching, respectively and the broad band from 3000 to 3600 cm^−1^ related to the O–H stretching vibrations, overlapped with NH_2_ stretching in the same region [33,34]. The FTIR spectra of viscose/cellulose and chitosan were fairly similar due to the structural similarities of both polymers. The spectra of differently pretreated viscose fabrics functionalized with chitosan show characteristic peaks for both polymers with slight modifications. The major bands of chitosan at 1560, 1590 and 1651 cm^−1^ corresponding to the stretching vibration of the amino group, N–H bending and carbonyl stretching of acetyl groups, respectively, almost disappeared, which could be explained by the fact that these absorption bands are in a region of the spectra where the broad band of adsorbed water masks their signal, but also by the formation of hydrogen bonds between viscose/cellulose and chitosan. Furthermore, the broad band between 3000–3600 cm^−1^, corresponding to the O–H stretching vibrations and NH_2_ stretching, was slightly shifted to higher wavenumbers and shows lower peak intensity, which confirms the formation of intermolecular hydrogen bonds between viscose and chitosan [35]. In the case of the TEMPO CV/CS sample, the band attributed to the C=O stretching vibration of the carboxylate anions, was slightly shifted to higher wavenumbers from 1600 to 1610 cm^−1^ and with lower intensity, which can be attributed to the interaction between positively charged ammonium groups of chitosan and the negatively charged carboxylate groups of TEMPO CV [36]. However, there was no evidence of a reaction between aldehyde groups from viscose and amino groups of chitosan, since the characteristic band (due to the covalent binding of chitosan by forming a Schiff base) was in a region of the spectra where the unavoidable absorbance of non-removable water bound to both polymers could mask its signal. The obtained results indicate that chitosan was physically attached to the pristine and differently pretreated viscose fabrics, while in the case of TEMPO CV/CS electrostatic interactions (ionic binding) between chitosan and TEMPO CV were confirmed.

Furthermore, the elemental analysis was then employed to determine the chitosan content in the functionalized pristine and pretreated viscose fabrics, which was calculated from the nitrogen percentage of the nitrogen originating from chitosan. The results show that, compared to the functionalized pristine viscose fabric binding 0.77 g chitosan/100 g cellulose, the pretreatment increased the ability of viscose fabric to bound chitosan (1.78 g chitosan/100 g cellulose and 1.13 g chitosan/100 g cellulose for TEMPO CV/CS and TOCN CV/CS, respectively), i.e., the chitosan content increased in the functionalized pretreated viscose fabrics for 131% in the case of previous TEMPO oxidation, and for 47% in the case of viscose fabrics previously coated with TOCN. In a certain way, the obtained results might be explained by new binding sites for chitosan, yielded onto the fiber/fabric surface with both pretreatments (Table 2, right column).

#### 3.1.3. Electrokinetic Properties of Viscose Fabrics

**Figure 2:**
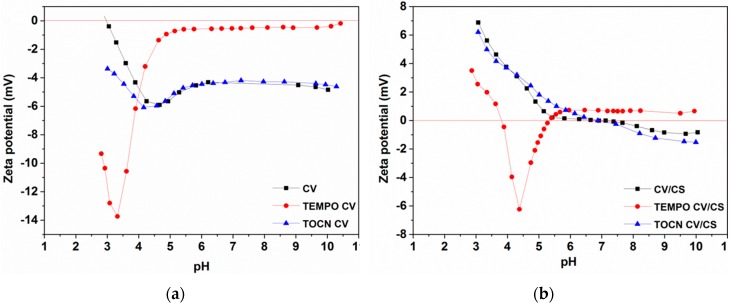
Zeta potential of pristine and pretreated viscose fabric (**a**) before and (**b**) after chitosan functionalization.

Surface charge is a crucial parameter for enhancement or suppression of the interaction between dissolved compounds in an aqueous solution and solid material surfaces. The zeta potential (ζ), used primarily as the indicator for solid surface charge, is a valuable parameter for the comparison of material surfaces before and after surface treatment, as well as their charging behavior in an aqueous solution [37]. Figure 2 presents pH-dependent zeta potential curves of pristine and pretreated viscose fabrics before and after functionalization with CS. Pristine and both pretreated viscose fabrics exhibit negative zeta potential throughout the entire pH region due to the presence of carboxyl and hydroxyl groups in the cellulose polymer (Figure 2a). The isoelectric point (IEP) shift from 2.94 for pristine to 1.74 for TEMPO oxidized, and to 1.81 for TOCN coated viscose fabrics, was a consequence of increased acidic (carboxyl) group content on the fabric surface. Although the carboxyl group content was significantly higher for TEMPO oxidized viscose compared to TOCN coated viscose (Table 2), its IEP was just slightly lower, indicating that in the case of TEMPO oxidized viscose the introduced functional groups were uniformly distributed into/onto the fabric/fibers, while in the case of the TOCN coated viscose these groups were distributed only on its surface. Furthermore, considering the fact that the plateau value at basic pH should be more negative in the case of higher carboxyl group density at the fabric surface, the obtained, less negative zeta-potential plateau value for the TEMPO oxidized viscose fabric (which has a higher carboxyl group content than pristine and TOCN coated viscose) can be explained by the fact that charged surface groups (i.e., deprotonated carboxyl groups) might prevent the dissociation of neighboring (carboxyl) groups by electrostatic repulsion, as well as by its increased hydrophilicity and swelling. With enhanced swelling, the ratio between the maximum negative zeta potential (at a slightly acidic pH) and the plateau value (at a high pH), ζ_max_/ ζ_plateau_, increases [28]. Unlike with the pristine and TOCN coated viscose, the decrease of the negative zeta potential at a high pH and an increase of the ζ_max_/ζ_plateau_ ratio for TEMPO oxidized viscose indicated its increased hydrophilicity and swelling as a consequence of changes in the chemical composition and structure caused by TEMPO oxidation. Such changes result in an enhanced accessibility of the inner fiber surface, as well as an increased total fiber surface area, especially in the swollen state, increasing its capacity for binding different ions or bioactive molecules [38,39].

Successful functionalization of viscose fabrics with CS is evident from the changes in the zeta potential–pH curves of pristine and pretreated viscose fabrics after the adsorption of chitosan from solution (Figure 2b). The zeta potential–pH curves for the pristine and TOCN coated viscose fabrics after functionalization with CS demonstrated that these fabrics exhibited typical amphoteric behavior owing to the introduction of CS amino groups onto their surface. The IEPs were shifted to a higher pH region (around pH 7) and the whole zeta potential–pH curves were shifted to less negative values in the higher pH region, and to positive values in a lower pH region. The less negative zeta potential plateau values implied a lower number of residual accessible carboxyl groups on the fiber/fabric surface. The amount of residual carboxyl groups depended on the intensity of ionic interaction with amino groups. At lower pH conditions, the surface was positively charged due to suppressed dissociation of free acidic groups from cellulose and enhanced protonation of free amino groups from CS. In the case of TEMPO oxidized viscose, its IEP was also shifted to a higher pH region (pH 4), while the whole zeta potential–pH curve was shifted to higher values due to a lower number of residual accessible acidic groups on the fiber/fabric surface. The zeta potential–pH curve was shifted to positive values in a higher pH region, but to less negative values in a lower pH region. The less negative values of zeta potential implied that the surface of the TEMPO oxidized viscose fabric after functionalization with CS was not completely covered with CS, i.e., on the surface of the viscose fabric, besides the CS amino groups, there were also free acidic groups from cellulose, which were accessible for dissociation at an acidic pH. On the other hand, in the alkaline pH range the protonation of CS amino groups should be suppressed (IEP of CS was about 6.8 [40]) and negative zeta potential of the TEMPO oxidized viscose fabric after functionalization with CS is expected. The slightly positive values of zeta potential obtained in a higher pH region could be, according to literature [41], explained by charge reversal, attributed to strongly adsorbed sodium ions, which were present in the chitosan solution used for functionalization, but also as counter ions to carboxylate groups in the TEMPO oxidized viscose. 

#### 3.1.4. Surface Morphology of Viscose Fabrics

**Figure 3:**
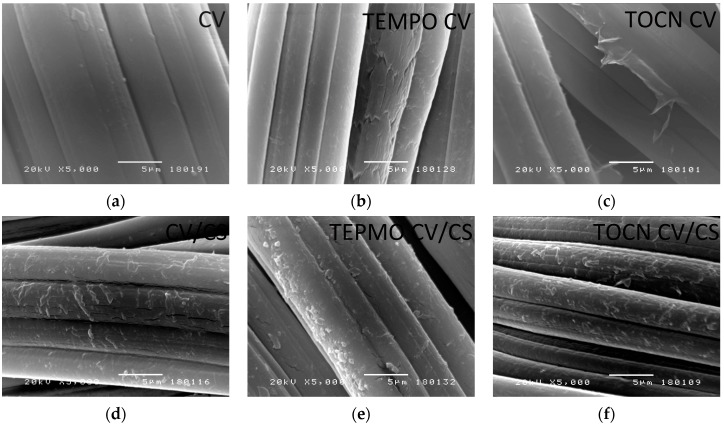
SEM images of pristine viscose (CV) (**a**), TEMPO oxidized CV (TEMPO CV) (**b**), TOCN coated CV (TOCN CV) (**c**), chitosan coated CV (CV/CS) (**d**), chitosan coated TEMPO oxidized CV (TEMPO CV/CS) (**e**) and chitosan coated TOCN CV (TOCN CV/CS) (**f**).

The morphology of the fibers/fabrics surface was assessed by SEM analysis. The SEM images of the pristine and pretreated viscose fabrics before and after functionalization with CS are shown in Figure 3. Unlike the pristine viscose (Figure 3a), the TEMPO oxidized viscose fibers (Figure 3b) had a more open-surface due to the structure damages in the form of cracks on the fiber surface [39]. The latter could be explained as a consequence of the oxidation process, which results in an efficient immobilization surface offering anchoring points for an enhanced attachment of CS molecules. In the case of TOCN coated viscose (Figure 3c), TOCN formed a thin polymer film embedding the fibers in a polymer coating, as we discussed in our previous work [14]. However, patches of polymer coatings detached from the fiber surfaces can be seen. The influence of the surface chemistry and topography on the degree of adsorption and the structure of the formed CS coatings can be seen through the changes in the surface morphology of the CS coated fibers (i.e., fiber surface roughness, size and density of CS particles), as shown in Figure 3d–f. The CS molecules formed the laminar particles on the surface of pristine and pretreated viscose fibers. The surface of the pretreated viscose fibers dictated a more efficient adsorption, which, in the case of the TEMPO oxidized sample, was characterized by more densely packed particles. SEM images of the surface of pristine and pretreated viscose fibers confirmed that the treatments conducted prior to the functionalization with chitosan influence the morphology of the resulting functional coatings.

#### 3.1.5. Mechanical Properties of Viscose Fabrics

**Figure 4:**
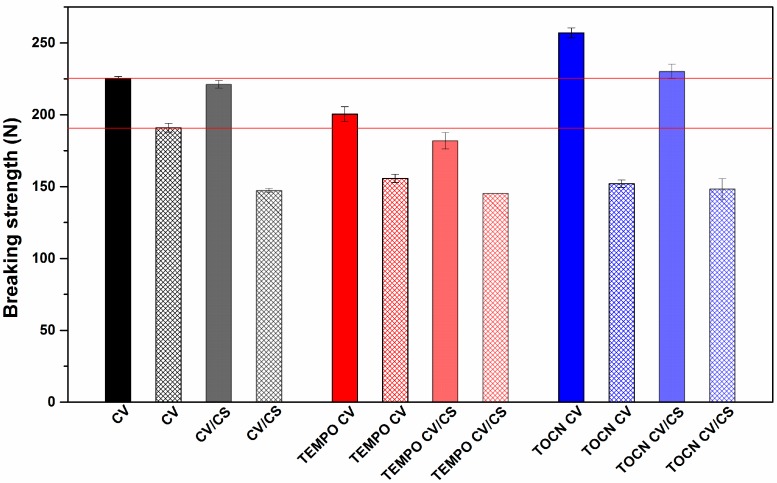
Breaking strength of pristine and pretreated viscose fabrics before and after chitosan functionalization: In warp (solid fill) and in weft direction (pattern fill).

Both oxidation and CS functionalization at an acidic pH remarkably reduce the mechanical properties of cellulose materials, which are directly related to the applicability of these materials [4,16,17,18,42,43]. Figure 4 shows the effect of different pretreatments and CS functionalization on the breaking strength of the pristine and pretreated viscose fabrics before and after functionalization with CS. In the case of TEMPO oxidation, along with the changes at the molecular level in terms of different types and quantity of the functional groups, the complex structure of cellulose fibers also changed, resulting in a decrease in breaking strength by 11% in warp, and by 2.5% in the weft direction. On the other hand, coating viscose with TOCN did not cause alterations in the cellulose supramolecular structure; the reinforcing effect of TOCN on the fibers’ surface can be clearly seen, i.e., the breaking strength of the TOCN CV sample increased by 14% in warp, and by 23% in the weft direction. Functionalization with CS was generally followed by a slight decrease in the breaking strength. This could be related to chitosan’s interactions with cellulose that were obviously happening in the inner part of the fibers, affecting their fibrillar structure, but also to the influence of acidic conditions used during the CS functionalization of the cellulose molecules [44].

#### 3.1.6. Antibacterial Properties of Viscose Fabrics

The antibacterial properties of functionalized viscose fabrics were evaluated against Gram-positive (*S. aureus*) and Gram-negative (*E. coli*) bacteria. In accordance with the used standard ASTM E2149-01, the sample shows antibacterial activity if the bacterial reduction was greater than 75%. The results presented in Table 3 indicate an efficient antimicrobial reduction by fabric samples functionalized with chitosan. A maximum reduction of *S. aureus* was evident for all investigated samples. In the case of *E. coli,* only the TEMPO CV/CS sample provided the highest reduction, while pristine and TOCN coated fabrics functionalized with CS provided a similar, excellent antimicrobial activity. The reduction results were almost the same; however, small changes might be explained by the highest content of attached chitosan in the case of the TEMPO CV/CS sample (Table 3), and consequently by accessible amino groups. It is known that the protonated amino groups in chitosan’s structure played a crucial role in its antibacterial activity. Actually, three antibacterial mechanisms of CS have been proposed: (1) The ionic surface interaction resulting in wall cell leakage; (2) the inhibition of the mRNA and protein synthesis via the penetration of CS into the nuclei of the microorganisms and (3) the formation of an external barrier, chelating metals and provoking the suppression of essential nutrients to microbial growth [45,46]. It is likely that all mechanisms occurred simultaneously but at different intensities.

It is important to mention that the achieved antibacterial activity against both tested stains was much higher than that reported for similarly functionalized viscose [1,9,11,46], for which sufficient antibacterial activity only against *S. aureus* was reported. However, antibacterial activity against both strains (*S. aureus* and *E. coli*) was reported in the case of viscose fabrics coated with chitosan-encapsulated iodine [9] and chitosan–curcumin formulations [5], as well as in the case of functionalization with water-soluble chitosan derivative N,O-carboxymethyl chitosan [7].

### 3.2. Washing Durability of Chitosan Functionalized Viscose Fabrics

Washing durability of CS functionalized viscose fabrics was monitored through changes in their electrokinetic surface properties, chitosan content and antibacterial activity after multiple washing, i.e., one, three and five washing cycles.

**Figure 5:**
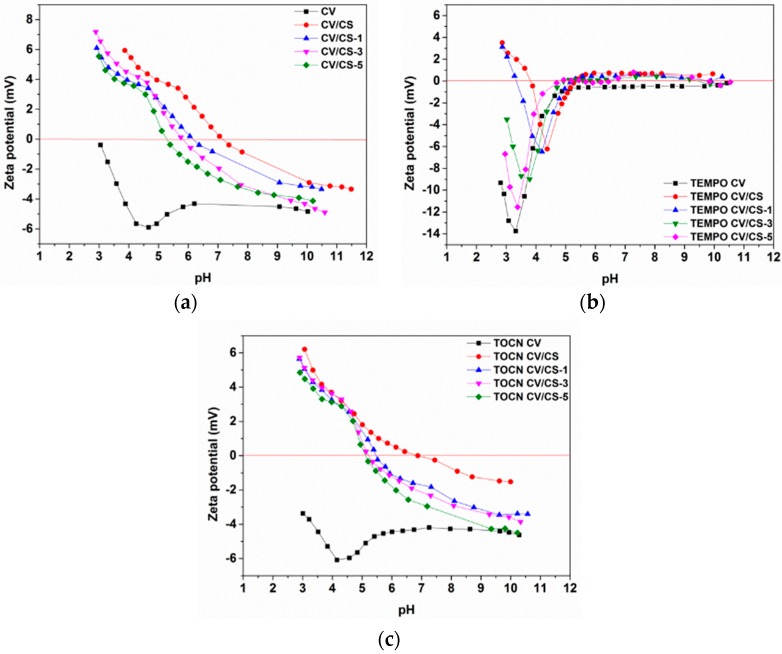
Zeta potential of pristine (**a**), TEMPO oxidized (**b**) and TOCN coated (**c**) viscose fabrics functionalized with chitosan before and after one, three and five washing cycles.

The changes of the electrokinetic surface properties of pristine and pretreated viscose fabrics functionalized with CS before and after multiple washing cycles can be seen in Figure 5. For all samples, after each washing cycle the zeta potential–pH curves were phase-shifting to a lower pH, i.e., to a lower IEP, which indicates a release of reversibly bounded CS molecules during washing. Comparing the zeta potential–pH curves of pristine and pretreated viscose fabrics before functionalization with CS and the CS functionalized fabrics after five washing cycles, it was clear that CS was still present on the fabrics’ surfaces.

**Figure 6:**
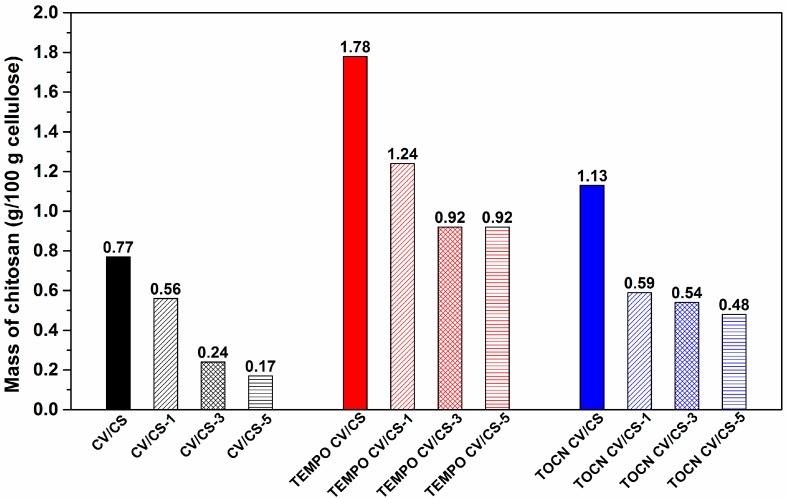
The chitosan content in the chitosan functionalized viscose fabrics after one, three and five washing cycles.

Compared to the pristine fabric, the pretreatments increased the stability of bounded CS during washing, and consequently its amount in the fabrics after five washing cycles. Taking into account the chitosan content determined after one, three and five washing cycles (Figure 6), the decrease observed after one washing cycle was 27% for pristine, 30% for TEMPO oxidized and 48% for TOCN coated viscose. An additional decrease in the chitosan content was observed after three washing cycles for all samples, most evident (about 57%) for the CS functionalized pristine viscose (CV/CS–3). After five washing cycles, again, the highest additional decrease in the chitosan content, of about 29%, was observed for CS functionalized pristine viscose, which shows the poorest washing durability, with a total decrease in chitosan content of about 78%, after five washing cycles, and the lowest chitosan content (0.17 g/100 g cellulose). TOCN CV/CS shows better washing durability, with a total decrease in the chitosan content of about 57% and a higher chitosan content (0.48 g/100 g cellulose) after five washing cycles. The best washing durability was evident for the TEMPO CV/CS sample; after three washing cycles, the total decrease in the chitosan content was about 48%, which left 0.92 g of chitosan/100 g cellulose, with no changes with further washings. The observed decrease in the chitosan content could be explained by the fact that a certain amount of chitosan was reversibly bound onto the cellulose surface [14].

**Figure 7:**
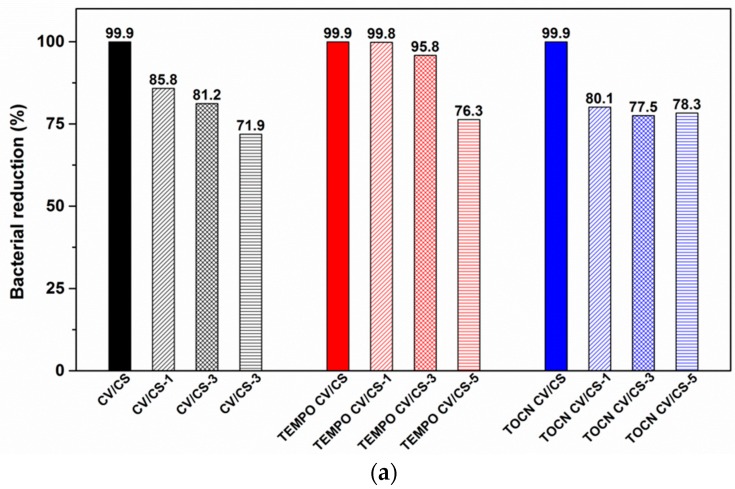

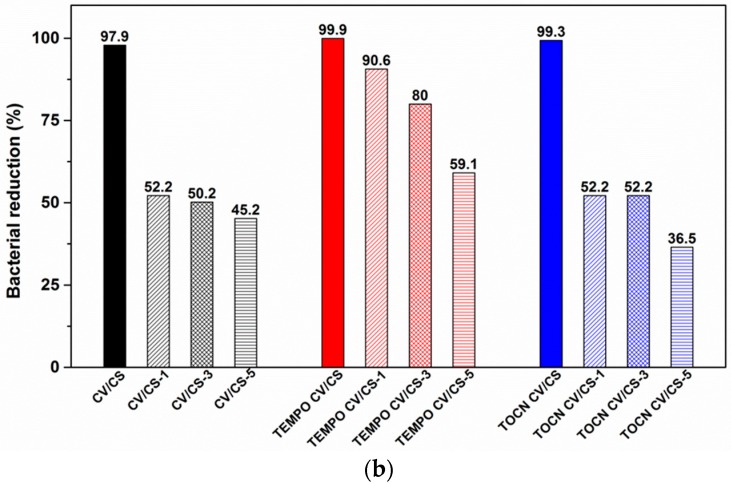
Bacterial reduction of pristine and pretreated viscose fabrics functionalized with chitosan after one, three and five washing cycles against *Staphylococcus aureus* (**a**) and *Escherichia coli* (**b**).

Moreover, the antibacterial activity of chitosan functionalized viscose fabrics against Gram-positive (*S. aureus*) bacteria was confirmed for all samples after one, three and five washing cycles (Figure 7a), while in the case of Gram-negative (*E. coli*) bacteria, the antibacterial activity diminished with an increased number of washing cycles (Figure 7b). Regarding the mentioned, better activity of chitosan functionalized viscose against Gram-positive (*S. aureus*) compared to Gram-negative bacteria (*E. coli*), it can be explained by the presence of an outer membrane in Gram-negative bacteria, which acts as a barrier towards environmental impact, which is already reported [46].

The measured amounts of bounded CS and the demonstrated antibacterial activity after washing indicate that achieving a washing durable antibacterial activity of chitosan functionalized viscose was a great challenge because of two rather contradictory conditions, which have to be fulfilled: (1) A satisfactory amount of anchoring sites into/onto the viscose, onto which a required amount of CS could be bound irreversibly, and, at the same time (2) a required amount of accessible free amino groups of CS, which would be responsible for antibacterial activity. CS functionalized pristine viscose fabric provided effective bacterial reduction against *S. aureus* up to three washing cycles, and against *E. coli* only before washing. This suggests that physical interactions between viscose and CS provide an additive effect strong enough to assure a sufficient amount of both bonded CS and accessible free amino groups of CS, which provides antibacterial activity before washing and washing durable antibacterial activity against *S. aureus*. CS functionalized TOCN coated viscose fabric provided effective bacterial reduction against *S. aureus* even up to five washing cycles, while against *E. coli* the effect was present only before washing, similar to pristine viscose fabric. Compared to the pristine, the TOCN coated viscose showed slightly lower antibacterial activity against *S. aureus* after one and three washing cycles regardless of the higher chitosan content remaining after corresponding washing cycles (Figure 6). These can be explained by higher surface charge density of chitosan functionalized pristine viscose in an acidic pH region (Figure 5) due to more accessible free amino groups. Comparing the results obtained for all tested samples, it was evident that CS functionalized TEMPO oxidized viscose showed the most effective and durable antibacterial activity against both types of bacteria i.e., effective bacterial reduction against *S. aureus* for up to five washing cycles and *E. coli* for up to three washing cycles. In general, TEMPO oxidation and coating with TOCN resulted in a higher degree of CS adsorption into/onto fabrics, as well as its better stability during washing and consequently higher amount of CS in fabrics after washing (Figure 6). Furthermore, both CS functionalized, pretreated fabrics showed more effective and durable antibacterial activity in comparison to CS functionalized pristine viscose fabric (Figure 7), which can be explained by a higher amount of accessible free amino groups of CS (Figure 5).

## 4. Conclusions

In this study, two different pretreatments of viscose fabric, TEMPO oxidation and coating with TOCN, were used to improve viscose fabric functionalized with CS, by introducing carboxyl and aldehyde groups into/onto viscose fabric for irreversible binding of CS. Both pretreatments contribute to the increase in the content of carboxyl and aldehyde groups, and thus the ability of the viscose fabrics to adsorb CS. As a consequence of a higher amount of irreversibly bound CS, pretreatments contribute to more efficient and durable antibacterial activity. Chitosan functionalized TEMPO oxidized viscose showed the most durable antibacterial activity against both bacteria (*S. aureus* and *E. coli*). However, TEMPO oxidation deteriorates mechanical properties of viscose fabric, while coating the viscose with TOCN improves them. Keeping that in mind, this chitosan functionalized fabric, obtained through the presented novel pretreatment for improving properties of textile materials, i.e., coating the viscose fabric with TOCN and its subsequent CS functionalization, is a high value-added product whose antibacterial and mechanical properties are simultaneously improved, and presents a good starting point for creating an upgraded nanostructure that would achieve an even greater improvement of both properties. Owing to the obtained antibacterial properties, the CS functionalized pristine viscose and both pretreated viscose fabrics do have a very high potential to be applied in manufacturing washable antibacterial textiles intended for people with sensitive skin prone to wounds, and a vast number of healthcare and hygiene products for use in the operating theater or in hospital wards for the hygiene, care and safety of staff and patients.

## Figures and Tables

**Table 1:** Notation of the prepared samples.

Description of Samples	Washing Cycles			
	0	1	3	5
Pristine viscose	CV	–	–	–
TEMPO oxidized viscose	TEMPO CV	–	–	–
Viscose coated with TOCN	TOCN CV	–	–	–
CV + chitosan solution	CV/CS	CV/CS–1	CV/CS–3	CV/CS–5
TEMPO CV + chitosan solution	TEMPO CV/CS	TEMPO CV/CS–1	TEMPO CV/CS–3	TEMPO CV/CS–5
TOCN CV + chitosan solution	TOCN CV/CS	TOCN CV/CS–1	TOCN CV/CS–3	TOCN CV/CS–5

**Table 2:** Content of functional groups of TEMPO oxidized cellulose nanofibrils (TOCN), pristine and pretreated viscose fabrics.

Sample	COOH, mmol/kg	CHO, mmol/kg	COOH + CHO, mmol/kg
CV	64	18	82
TEMPO CV	438	440	878
TOCN	830	90	920
TOCN CV	86	27	113

**Table 3:** The bacterial reduction of chitosan functionalized viscose fabrics.

Sample	Bacterial Reduction, %	
	*Staphylococcus aureus*	*Escherichia coli*
CV/CS	99.9	97.9
TEMPO CV/CS	99.9	99.9
TOCN CV/CS	99.9	99.3
